# Extremely high canine C-reactive protein concentrations > 100 mg/l – prevalence, etiology and prognostic significance

**DOI:** 10.1186/s12917-020-02367-7

**Published:** 2020-05-20

**Authors:** Sarah Hindenberg, Natali Bauer, Andreas Moritz

**Affiliations:** Department of Veterinary Clinical Sciences, Clinical Pathology and Clinical Pathophysiology, Justus-Liebig-University Giessen, Frankfurter Str. 114, 35392 Giessen, Germany

**Keywords:** Acute phase protein, Canine, Dog, CRP, Inflammation, Bacterial, Decision limit, Prevalence

## Abstract

**Background:**

In human medicine, extremely high CRP (C-reactive protein) concentrations > 100 mg/l are indicators of bacterial infection and the need of antibiotic treatment. Similar decision limits for septic pneumonia are recommended for dogs but have not yet been evaluated for other organ systems. The aim of the retrospective study was to investigate the prevalence and evaluate dogs with CRP concentrations > 100 mg/l regarding the underlying etiology, the affected organ system and the prognostic significance.

**Results:**

Prevalence of CRP > 100 mg/l was investigated in dogs presented between 2014 and 2015 and was 12%.

For evaluation of etiology and organ systems, dogs with CRP > 100 mg/l presented between 2014 and 2016 were enrolled. Dogs were classified into 4 main disease categories, i.e. inflammatory, neoplastic, tissue damage or “diverse”. Diseases were assigned to the affected organ system. If an organ classification was not possible, dogs were classified as “multiple”. 147 dogs with CRP 101–368 mg/l were included and classified into disease categories: 86/147 (59%) with inflammatory etiology (among these, 23/86 non-infectious, 44/86 infectious (33/44 bacterial), 19/86 inflammation non-classifiable), 31/147 (21%) tissue damage, 17/147 (12%) neoplastic (all malignant) and 13/147 (9%) diverse diseases. The affected organ systems included 57/147 (39%) multiple, 30/147 (20%) trauma, 21/147 (14%) gastrointestinal tract, 10/147 (7%) musculoskeletal system, 8/147 (5%) respiratory tract, 7/147 (5%) urinary/reproductive tract, 6/147 (4%) skin/subcutis/ear, 6/147 (4%) central/peripheral nervous system and 2/147 (1%) heart. The disease group (*p* = 0.081) or organ system (*p* = 0.17) did not have an impact on CRP. Based on CRP, a detection of bacterial infection was not possible.

The prognostic significance was investigated by determining the 3-months survival and hospitalization rate in a subgroup with known outcome. The 3-months survival rate was 46/73 (63%) while the majority 66/73 (90%) of patients was hospitalized.

**Conclusions:**

CRP concentrations > 100 mg/l are occasionally seen in a clinic population. They indicate a severe systemic disease of various etiologies with guarded prognosis. Extremely high CRP concentrations do not allow a conclusion of the underlying etiology or an identification of bacterial inflammation.

## Background

Acute phase proteins (APP) are sensitive markers that change their concentration as a reaction to a systemic inflammatory process [1] and are known to increase in response to infectious diseases [2–4], immune mediated diseases [5–7], neoplasia [8–10] and surgery [11]. APPs react more rapidly and with a shorter half-life period than classic markers of inflammation. C-reactive protein (CRP) is an important major APP in dogs, which increases within the first 8-24 h after an inflammatory stimulus [12, 13] and reaches up to 100-fold of the baseline levels [1]. This wide range permits a more detailed evaluation of an inflammatory process than leukocyte counts. CRP measurement is meanwhile widely available in veterinary medicine. According to state-of-the-art scientific research, the CRP range of healthy dogs is below ~ 10–20 mg/l CRP [12, 14, 15]. Canine CRP value increases of up to > 900 mg/l are reported in extremely rare cases [13, 16]. Different cut-off values have been discussed as medical decision limits for human and canine CRP [17]. While there is no general definition, which medical decision limits should be utilized to classify an inflammation as low grade or moderate, there is a consent that CRP values above 100 mg/l indicate a high grade inflammation [18–20]. While current research provides at least some information on extreme leukocytosis in small animals [21, 22], studies addressing extremely high CRP values in dogs are rare [3]. Scarce knowledge is available about the prevalence of CRP values above 100 mg/l in dogs. In humans, prevalence of CRP values > 100 m/l is ranging between 3 and 30% depending on the patient population [18, 23].

For humans, some inflammatory disease etiologies have proven to present with extremely increased CRP values more often than others [23]. To the authors’ knowledge, comparable information is scarce in dogs. However, it is well known for leukemoid reaction that such extreme leukocytosis is linked to only a small group of disease categories [21, 22]. Thus, it can be hypothesized that similar might be true for CRP.

Another critical point in a patient with a severe inflammation of unknown origin is the question as to whether antibiotic treatment is required, especially in times of increasing antibiotic resistances of bacteria [24]. Although acute-phase-proteins are relatively unspecific markers of different types of inflammation in human as well as in veterinary medicine [25], extremely high CRP concentrations > 100 mg/L in association with specific symptom complexes (e.g. signs of pneumonia or signs of meningitis) are indicative of bacterial inflammation [20, 26, 27] and thus a decision criterion for antibiotic treatment in human medicine. In dogs, the use of decision limits for CRP concentrations to recognize potential bacterial etiology have been rarely investigated. To the authors’ knowledge there is only one study in dogs with respiratory diseases that recommended a similar decision limit for detection of septic pneumonia as in humans [3]. However, the decision limit might not be the same for other organ systems. Moreover, dogs with neoplastic diseases had been ruled out in the previous study so that it can only be used in a very limited, rather artificial, setting.

Furthermore, extreme leukocytosis is associated with a high mortality and therefore a negative prognosis [22]. It is therefore a question of interest if single very high CRP values imply a negative prognosis for canine patients.

Our study was thus aimed to retrospectively evaluate dogs with extremely high CRP concentrations > 100 mg/l to answer the following questions:

1. How is the prevalence of such extremely high CRP concentrations?
2. Are they linked to certain disease categories or organ systems and what is the prognosis?
3. Do they indicate the necessity of antibiotic treatment?

Our hypothesis was that extremely high CRP concentrations are indicative of severe diseases associated with a grave prognosis, however, they cannot predict the necessity of antibiotic treatment.

## Results

### Prevalence of CRP > 100 mg/l

Overall, 2184 CRP measurements of 1578 dogs were included. In 225/2184 analyses (10.3%), CRP concentration > 100 mg/l was detected. After removal of multiple measurements, median CRP concentration seen in 1578 dogs was 4 mg/l (range 0–363 mg/l. In 194/1578 dogs (12.3%), a CRP concentration > 100 mg/l (median 151 mg/l, range 101–363 mg/l) was demonstrated.

### Study population of dogs with CRP > 100 mg/l

Overall, 147 dogs were included in the study of disease groups and organ systems. In a subgroup of 73/147 dogs with known 3-months outcomes, the prognostic evaluation was performed. Seventy-six of the 147 dogs which were enrolled in the study were male and 71 were female. Among them were 49 mixed-breed dogs and 98 dogs of 33 breeds. The breeds were represented by 1–6 dogs each. The median age was 7 years. CRP values within a range of 101–368 mg/l were documented.

### Classification of diseases

#### Etiology

**Fig. 1:**
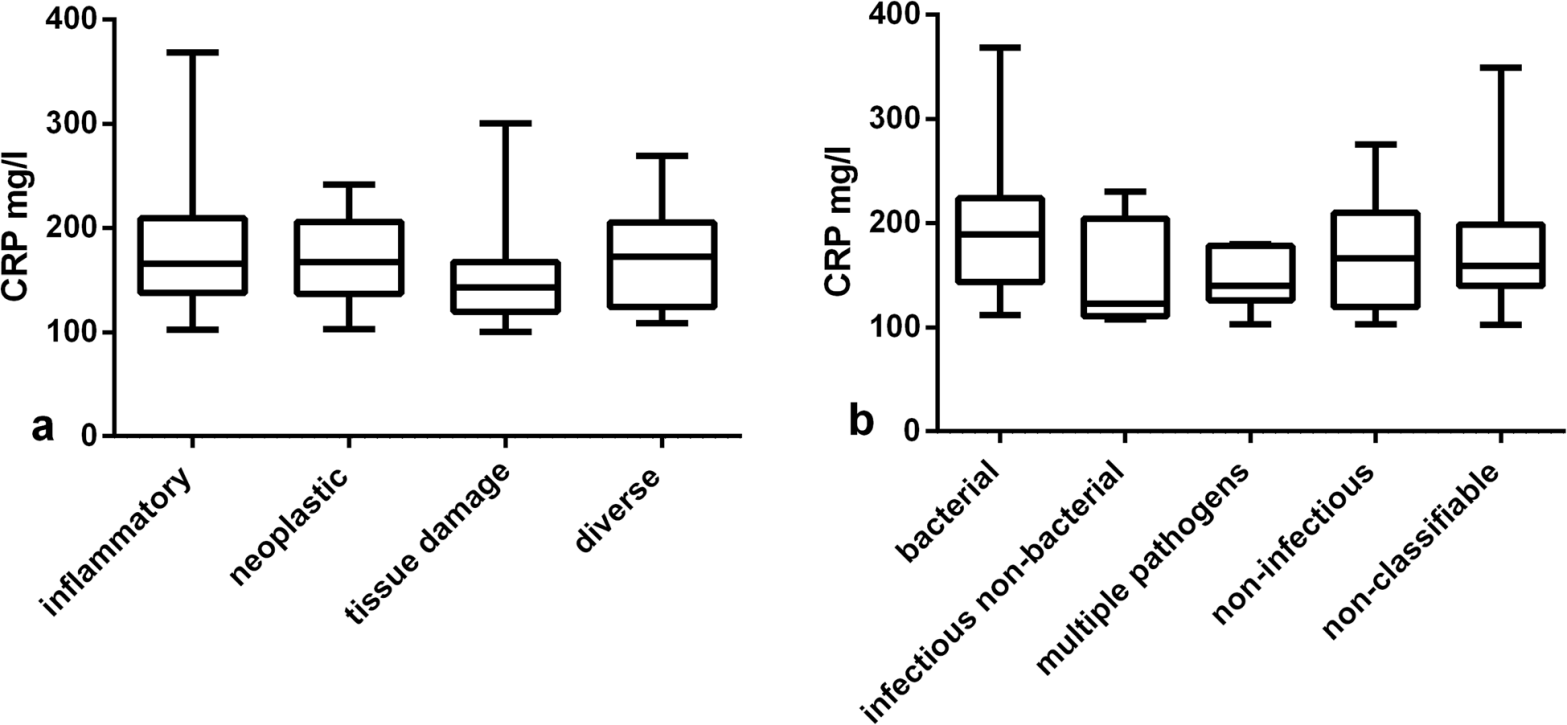
CRP concentration in dogs with CRP > 100 mg/l according to the disease category (**a**) as well as detailed presentation of the subcategories in dogs with inflammatory diseases (**b**). Results are shown as box and whisker diagrams. The horizontal line in the boxes is consistent with the median, the whiskers indicate the range and the box represents the 25th-75th percentile. The median values and inter quartile ranges (IQR) of the groups (median /IQR in mg/l) presented as follows: (**a**) inflammatory (165.6 /137.8–209.0), neoplastic (167.2 /137.3–205.7), tissue damage (143.0 /119.9–167.2), diverse (172.1 /108.7–205.0); (**b**) bacterial (189.2 /143.4–223.8), infectious non-bacterial (122.8 /110.8–204.0), multiple pathogens (139.7 /126.2–178.2), non-infectious (166.3 /119.4–209.8), non-classifiable (158.9 /139.8–198.6)

The dogs were classified into disease categories as follows (Fig. 1a-b): 86/147 (59%) with inflammatory etiology, 17/147 (12%) neoplasia, all of them malignant, 31/147 (21%) tissue damage and 13/147 (9%) diverse diseases.

The “inflammation” group was further subdivided into 23/86 (27%) non-infectious, 44/86 (51%) infectious and 19/86 (22%) non-classifiable.

The “infection” group was further subclassified as 33/86 (38%) bacterial, 4/86 (5%) infectious non-bacterial and 7/86 (8%) multiple pathogens.

Of the dogs classified as “bacterial infection”, 25/33 had a microbiological analysis, but only 14/33 a positive result. Two more dogs (2/33) had a cytological finding of bacteria or a finding highly suspicious for a bacterial infection respectively. The remaining 17/33 dogs were classified as bacterial infection according to the diagnostic findings and the clinical course: 4x intestinal foreign body, 3x pneumonia, 3x fever of unknown origin but responsive to antibiotics, 1x meningitis responsive to antibiotics, 1x gastroenteritis with signs of sepsis, 1x wound infection and 1x prostatic abscess.

No healthy dogs showed CRP values > 100 mg/l. Overall, about one fifth (22%) of all dogs were presented with bacterial infection.

About one third (27%) of the patients with inflammatory disease had diseases classified as non-infectious (Fig. 1b). Within this group, the majority of patients presented with immune-mediated diseases such as immune-mediated polyarthritis, steroid-responsive meningitis-arteritis, immune-mediated anemia or thrombocytopenia.

The solely malignant neoplasms were classified as epithelial 7/17 (41%), mesenchymal 7/17 (41%) and round cell neoplasms 3/17 (18%). Because of the small data set of round cell neoplasms, no further statistics were applied to investigate CRP values depending on the tissue origin of the type of neoplasia.

No statistical significance could be found between the median CRP concentrations of the different etiological groups (*p* = 0.081) or the inflammatory groups (*p* = 0.17). The median CRP concentration in the bacterial group was not statistically significantly different to other etiological groups, so that a discriminative cut-off value could not be determined.

#### Affected organ system

**Fig. 2:**
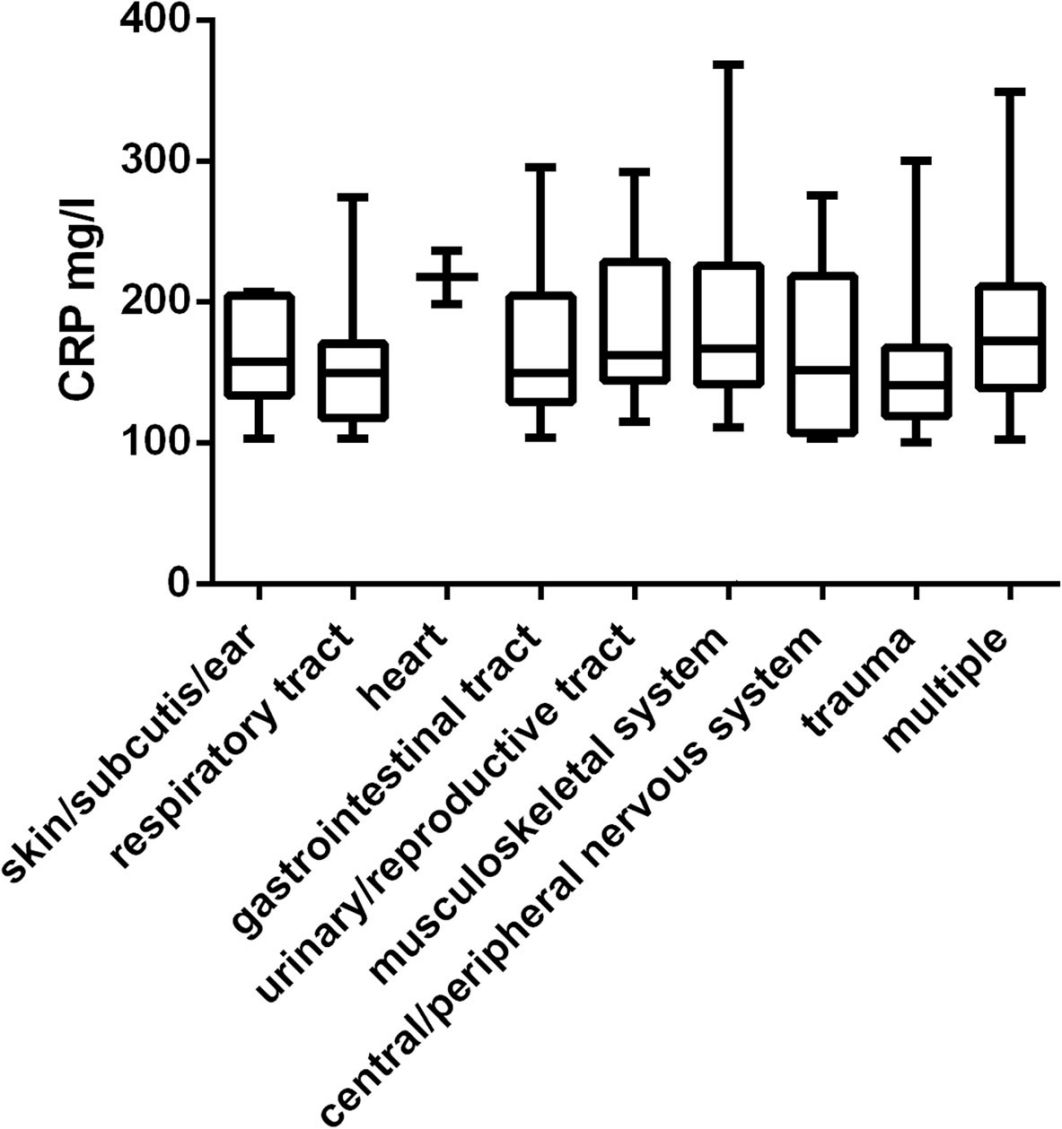
Influence of the organ system on the CRP concentration in dogs with CRP > 100 mg/l. The median values and inter quartile ranges (IQR) of the groups (median /IQR in mg/l) presented as follows: skin/subcutis/ear (157.6 /134.0–204.2), respiratory tract (149.5 /117.7–170.4), heart (217.4 /198.6–236.2), gastrointestinal tract (149.9 /129.0–204.2), urinary/reproductive tract (162.1 /144.0–228.2), musculoskeletal system (167.2 /141.9–225.4), central/peripheral nervous system (151.8 /107.1–218.2), trauma (141.2 /119.0–167.9), multiple (172.0 /139.0–210.8). For remainder of key, see Fig. 1

The organ system groups in which most often diseases with CRP > 100 mg/l were detected were: multiple 57/147 (39%), trauma 30/147 (20%), gastrointestinal tract 21/147 (14%) and musculoskeletal system 10/147 (7%), followed by respiratory tract 8/147 (5%), urinary/reproductive tract 7/147 (5%), skin/subcutis/ear 6/147 (4%), and central/peripheral nervous system 6/147 (4%). The smallest group was “heart” 2/147 (1%). The organ system did not have a significant impact on the median CRP concentration (*p* = 0.17, Fig. 2).

#### Prognosis

Overall, 46/73 (63%) of patients with known 3-months outcome and CRP concentrations > 100 mg/l survived, resulting in a mortality rate of 37%.

The highest mortality was seen in patients with neoplasia (8/10 died; 80%), followed by patients with diverse diseases (5/8; 63%), inflammatory diseases (12/46; 26%), and tissue damage (2/9, 22%), respectively. The mortality was markedly higher in non-infectious 42% (5/12) compared to infectious disease 13% (3/23), but the results have to be interpreted with caution as only small patient numbers were found in these subgroups.

For the vast majority of patients (66/73, 90%), a decision for hospitalization was made. Most (5/7, 71%) of the non-hospitalized patients had neoplastic diseases and were euthanized within the next two weeks.

**Fig. 3:**
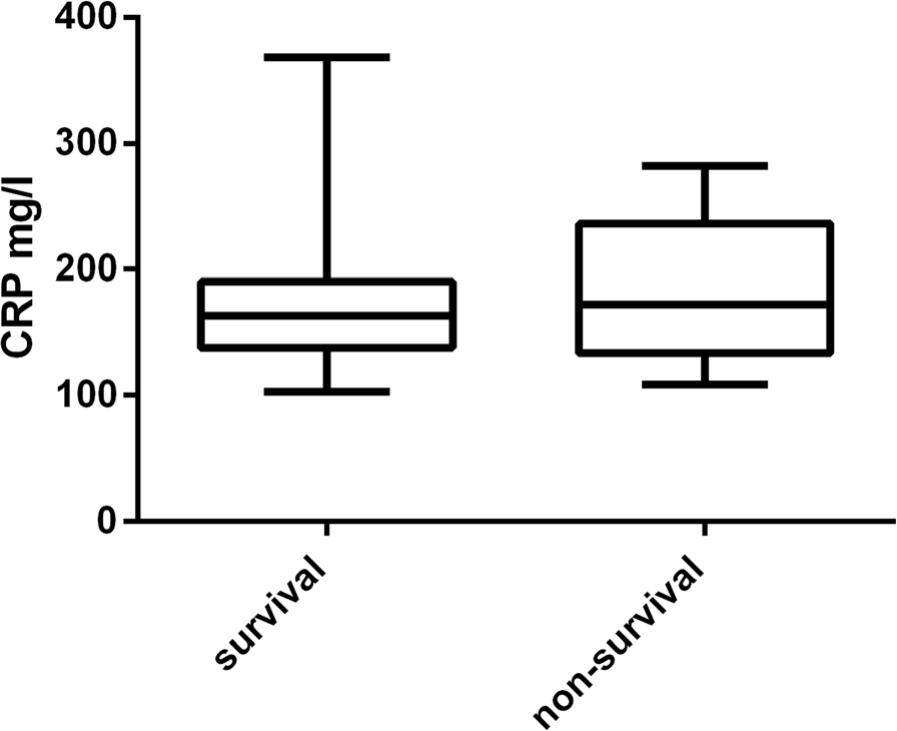
Impact of 3-months survival on CRP concentrations in dogs with CRP > 100 mg/l. The median values and inter quartile ranges (IQR) of the groups (median /IQR in mg/l) presented as follows: survival (163.5 /137.9–190.5), non-survival (172.1 /133.7–236.2). For remainder of key, see Fig. 1

**Fig. 4:**
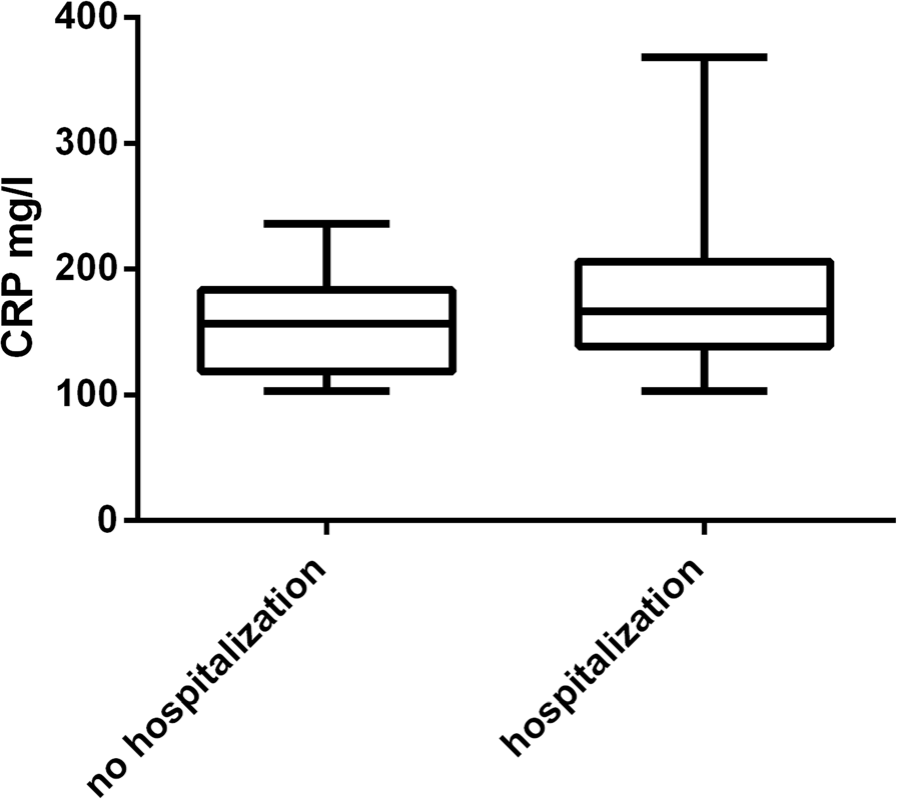
Impact of the need for hospitalization on CRP concentrations in dogs with CRP > 100 mg/l. The median values and inter quartile ranges (IQR) of the groups (median/IQR in mg/l) presented as follows: no hospitalization (156.4 /118.5–183.0), hospitalization (166.5 /138.4–205.8). For remainder of key, see Fig. 1

As seen in Figs. 3 and 4, 3-months-survival (*p* = 0.43) or the decision for hospitalization (*p* = 0.42) did not have an impact on CRP concentration in general.

## Discussion

Studies addressing extremely high CRP values in dogs are scarce [3] and are even rare in humans [18, 28]. To the authors’ knowledge, this is the first study evaluating extreme increases of CRP in a mixed canine patient population with various diseases. In our study, the decision limit for CRP was chosen in accordance with other human studies which often use cut-off values of 100 mg/l to rule in a bacterial infection and apply antibiotics.

### Prevalence of CRP > 100 mg/l

To the authors’ knowledge, there are no studies in dogs primarily aimed to investigate the prevalence of extremely high CRP values above 100 mg/l. However, when evaluating data reported previously for a mixed canine patient population in a veterinary university clinic in Japan, 9% dogs had a CRP > 100 that is in accordance with our findings [19]. In humans, reported prevalence for CRP > 100 mg/l is highly variable and ranges between 3 and 30% [19, 23]. The most likely explanation for the highly variable results is the patient population. While a low prevalence of CRP > 100 will be expected in primary health care facilities, a high prevalence is observed in hospitals with a large number of severely diseased patients.

### CRP cut-off to differentiate etiologies

Published human [26] and canine [12, 14, 15] reference ranges, including our own, for CRP measured with different assays are almost similar (< 10–20 mg/l) so that theoretically a similar behavior of both, human and canine CRP could be expected. While bacterial infections have been reported in 55% (462/839) [18] to 78% (35/42) [20] of human patients with CRP concentrations > 100 mg/l, only 22% (33/149) of canine patients in our study fell into this category. In contrast to human beings, high CRP values above 100 mg/l were not able to discriminate between different disease etiologies in dogs in our data set. Our data confirm the theory that CRP cannot be used as a differentiation parameter to distinguish between infectious or even bacterial and non-infectious /non-bacterial diseases in dogs. Therefore, high CRP values are no indication for antibiotic treatment. Extremely high CRP values indicate a severe inflammation with acute phase reaction with various etiologies. The proportion of non-infectious diseases seen here (12%) is comparable to a recent study in humans (rheumatologic diseases ~ 8%) [18]. In contrast, the proportion of neoplastic diseases (16%) is higher in our study compared to human medicine, where these patients only account for ~ 5% [18]. A descriptive study on dogs with various diseases included a high proportion of patients with immune-mediated diseases or cancer among dogs with > 100 mg/l CRP which is in accordance with our results [19].

As infectious etiologies were most often (55%) the reason for extremely high CRP values in humans, it is recommended to rule out infections as a first step of the diagnostic workup [18]. This could also be adopted to veterinary medicine, keeping in mind that especially non-infectious inflammations and neoplasia are also common etiologies. The fact that the diagnosis “bacterial infection” in our study was not always based on a positive microbiological finding might be seen as a limitation of the study. To our own experience and also according to recent research, microbiological analysis is helpful and important but not infallible. Negative results occur in patients with definite bacterial disease [29]. In human medicine, these are 25–60% of the samples [30]. It is acknowledged to start antimicrobial therapy in critically ill patients based on the suspicion of a bacterial infection [31]. In cases of negative cultural results, it is up to the clinician’s decision if antibiotics are necessary [31, 32]. On the other hand, positive microbiological results do not always prove an infection. There may be (pre) analytical problems such as contaminations or an overgrowth with bacteria that have not been the original pathogens. Furthermore, it is not always possible to take microbiological samples due to the anatomical region of inflammation or the critical state of the patient. Therefore, we took the conscious decision not only to include cases with (positive) bacteriological examination results in our study, but also to include cytological and histological findings as well as the clinical course of the patient. This is handled similarly in other studies [33].

In human and animal patients with neoplasia and in absence of an additional disease, major inflammatory reactions are also a characteristic of malignant tumors (e.g. mammary carcinomas, lymphatic neoplasms) [10, 34]. This is in accordance with our study, where CRP values > 100 mg/l were only found in association with malignant and not benign neoplasia. Benign neoplasia is seldom described to cause major inflammatory reactions [9] and might be associated with ulceration in these cases [10, 34]. In human medicine, a proportion of about 25–40% of patients with malignant cancer has associated infectious or non-infectious mild to severe inflammation, which may be accompanied by a systemic acute phase reaction [35–37]. A similar explanation might be true for canine patients in general [34] and for the marked acute phase reaction seen in cancer patients in our study. Nevertheless, diagnostics concerning a possible secondary bacterial infection are indicated in patients with tumor and high CRP values [8, 20].

Our study clearly showed that also trauma or surgery without evidence of bacterial infection might induce an extreme increase in CRP concentrations > 100 mg/l. It is well known that traumatic events lead to tissue damage and induce an acute phase reaction within the first 24 h that subsides gradually until the point of time of suture removal [11, 38, 39]. The increase of CRP does not only depend on the degree of trauma [16, 39] but may also depend on the CRP value before surgery, the surgeon and analytical variations [29]. Another limitation of our study, however, might be the fact that bacterial infections could not always be excluded especially if surgery was performed in primarily “non-sterile” organs such as the gastrointestinal tract. This may have led to misclassifications of the disease category.

### CRP cut-off to differentiate diseased organ systems

Our findings in dogs demonstrated that extreme increases in CRP cannot be attributed to specific organ systems. Nevertheless, it was obvious that some organ systems tend to be more frequently involved in marked inflammations. On the one hand, these are organ systems which are frequently exposed to bacteria (e.g. the gastrointestinal tract [19, 28, 40]), but on the other hand these are also organ systems which are affected by non-infectious, often immune-mediated, diseases (e.g. the musculoskeletal system [41, 42]). CRP analyses of patients with diseases of the reproductive organs are likely underrepresented in our study as they are primarily referred to the clinic for obstetrics. Interestingly, an organ system with frequent contact to microorganisms (respiratory tract), which is known to present with high CRP values [3, 28] in the case of bacterial infection, was underrepresented in our study. A reason for that could be that patients with bacterial pneumonia, seen in our university clinic, partly present with complicated disease progresses (e.g. pyothorax secondary to septic foreign body in the lung) or suffer from multiple diseases (aspiration pneumonia secondary to another disease, pneumonia due to immunosuppression) and were therefore not always classified as solely “respiratory patients”. Given our results, the attempt to define a cut-off value to detect septic inflammation is only promising for organ systems rarely affected by immune mediated diseases and after ruling out malignant neoplasia with high probability as it has been done previously for the respiratory tract [3].

### Prognostic value of CRP

A mortality rate of 37% found here, was markedly higher than reported in humans with CRP > 100 mg/l, where the overall mortality rate during hospitalization was ~ 9% [18]. The markedly lower mortality rate in humans can be explained by the fact that it was only assessed until the day of hospital release [18]. Moreover, the fact that the option of euthanasia is given in veterinary medicine, which leads to higher “mortality” for severely ill patients, has to be taken into account. The arguments (clinical condition, prognosis, financial reasons, management problems, emotional/ private constitution of the owner, and recommendation of the veterinarian) that led to euthanasia were not evaluated here, which is a limitation of the study. The influence of more advanced life-sustaining measures in human medicine might also have contributed to the better outcome reported in humans.

According to our data, there is a high mortality rate in canine patients (80%) as it is known for human cancer patients with CRP > 100 mg/l. However, it has to be taken into account that the mortality (10–40% in humans with CRP > 100 mg/l [18]; > 60% with CRP > 500 mg/l [28]) is also highly dependent on the underlying etiology.

In contrast, canine trauma patients with high CRP values have a comparatively good outcome as demonstrated here. Previous human studies did often not consider patients with traumatic disease etiology at all (tissue damage) [18, 28]. Moreover, there was also a relatively low mortality in patients with inflammatory diseases.

In various etiologies, CRP has proven to be a marker of disease activity or severity and therefore indirectly, a prognostic marker in dogs [40, 43, 44].

The absolute CRP values in our study are not statistically different for survivors compared to non-survivors. The same was described for initial CRP values in dogs with primary immune-mediated hemolytic anemia (IMHA) [45], Ehrlichiosis [4] and for a mixed population of critically ill dogs [46, 47]. CRP has been shown to be a marker of moderate diagnostic value reflecting disease severity in parvoviral enteritis [43, 48]. In contrast to our results and the previous veterinary studies, in human patients with severe sepsis, CRP was significantly higher in non-surviving patients [49] – surprisingly even without an overlap of the patient groups on the day of admission. Also data for the novel COVID-19 virus in humans imply a negative prognostic significance of high CRP values [50].

The most likely explanation why these results differ from our results is the study population. It appears to be logical that in a quite homogenous group with one disease etiology, higher CRP values indicate a higher severity of disease and worse prognosis. Furthermore, the diagnosis “systemic inflammatory response syndrome (SIRS)” is already an advanced state of disease, while in our mixed study population, dogs with different diseases and disease states are included. Additionally, it has to be taken into account that our study population was initially “biased” by the cut-off of 100 mg/l CRP, while other studies investigate the whole spectrum of CRP values [49]. In our study, evaluation of prognosis was only made in a subgroup of patients (patients with known prognosis according to the patient data system), potentially influencing the evaluation results as the clinic may lose contact due to different reasons (owner happy/unhappy, animal healthy/dead, practical reasons).

It has been shown that dogs with marked inflammation tend to need hospitalization inducing high costs for treatment [51]. The high hospitalization rate of 90% seen here, can be explained with the fact that the majority of dogs was clinically severely ill. Nevertheless, there was no association of CRP and hospitalization. Similar findings have been reported for dogs with autoimmune hemolytic anemia or pyometra [7, 52]. The lack of impact of hospitalization on a single CRP result > 100 mg/l demonstrates that the absolute CRP value alone is not a definite prognostic marker but rather indicates a severe disease. Five of the seven (71%) non-hospitalized dogs were cancer patients. In these patients, the decision against hospitalization was probably not made because of a mild disease but due to the unfavorable prognosis. All five dogs were euthanized within the next two weeks.

This study confirms that extremely high CRP concentrations are an indicator of severe systemic inflammation with acute-phase reaction but should not be seen as an indicator of bad prognosis with the consequence of euthanasia. Instead, the consequence has to be an early start of more advanced diagnostics and therapy as well as close monitoring. The actual prognosis for the individual patient is highly dependent on the disease etiology.

Our study was limited by its retrospective nature. A definite diagnosis up to one single detailed etiology could not be made in some cases. Patients were evaluated in different states of disease. Several patients were already pretreated. This is a known problem of medical studies investigating naturally diseased patients, especially if conducted in referral hospitals. In our study, especially the pretreatment with anti-inflammatory or antibiotic agents is a limitation which might have lowered CRP concentrations. Nevertheless, it can be assumed that canine patients presented to our clinic still had an active disease process, even if pretreated. It has been shown that antibiotic pretreatment in dogs with > 24 h respiratory symptoms did not significantly affect the CRP concentration as well as pretreatment with glucocorticoids [3, 6]. Moreover, underlying conditions might have an impact on CRP concentrations. It is known that severe liver failure might lower the CRP value, but only little information is available in literature [53]. Statistical evaluation was hampered by the relatively small patient number in some subgroups so that significances might have been missed.

## Conclusion

Extremely high CRP concentrations > 100 mg/l occur in about 12% of patients in a third opinion veterinary hospital and are indicative of a severe systemic disease with guarded prognosis and are observed due to various etiologies such as trauma, infection, immunopathy, and malignant neoplasia. However, such extreme increases in CRP do not allow a determination of the underlying etiology or a differentiation between bacterial and non-bacterial inflammation. The individual prognosis significantly depends on the specific underlying etiology. Further diagnostics are therefore indicated and the patient should be closely monitored.

## Methods

### Prevalence of dogs with CRP > 100 mg/l

Due to the large data set, prevalence of CRP > 100 was assessed for a smaller subcategory of data that allows to remove multiple measurements of dogs with reasonable effort. Consecutive laboratory results of dogs presented between April 2014 and April 2015 to the Clinic for Small Animals (Internal Medicine, Surgery), Faculty of Veterinary Medicine, Justus-Liebig-University, Giessen, Germany were included.

1. Prevalence of CRP > 100 mg/l among laboratory data
2. Prevalence of CRP > 100 mg/l in patients: In case of multiple measurements of the same patient with a CRP concentration < 100 mg/l, the first data set was taken. For dogs with CRP > 100 mg/l, the first data set showing CRP > 100 mg/l was included.

### Study population – dogs with CRP > 100 mg/l

Using the patient documentation system easyVET (VetZ GmbH, Isernhagen, Germany), data of dogs presented between March 2014 and December 2016 to the Clinic for Small Animals (Internal Medicine, Surgery), Faculty of Veterinary Medicine, Justus-Liebig-University, Giessen, Germany, were retrospectively included into the study if the following inclusion criteria were fulfilled:

1. Minimal database: hematological profile and clinical chemistry profile including a CRP value > 100 mg/l, measured in the Department of Veterinary Clinical Sciences, Clinical Pathology and Clinical Pathophysiology, Justus-Liebig-University, Giessen, Germany.
2. Documentation of the diagnostic process in the patient documentation system easyVET.

In case of repeated CRP measurements exceeding 100 mg/l in the same patient, the first value with minimal database was included.

As most patients presented to our clinic had received medication before, patients with premedication were not excluded if they were still symptomatic for the disease.

### Measurement of CRP

Laboratory analyses (hematology, clinical chemistry) were performed as part of routine diagnostics. CRP analyses were performed with lithium heparin plasma using the immunoturbidimetric species-specific Gentian Canine CRP Immunoassay (Gentian AS, Moss, Norway) on the ABX Pentra 400 clinical chemistry analyzer (ABX Horiba, Montpellier, France) containing polyclonal chicken-derived antibodies against canine CRP. The assay has been validated on different bench top analyzers before and has proven to be a reliable high quality option to measure canine CRP [15, 54]. A laboratory-intern reference interval for canine CRP was established based on serum samples obtained from 77 healthy adult (> 1 year old, median age 1 year, range 1–8 years) dogs presented at the Clinic for Small Animals, Faculty of Veterinary Medicine, Justus-Liebig-University, Giessen, Germany between February 2011 and June 2013 for routine radiologic examination to screen for hereditary hip or elbow dysplasia, as blood donors or for health checks. The one-sided 95% reference interval was calculated using the Excel-based software refence value Advisor 2.1 [55]. Prior to calculation of the reference interval, the Anderson-Darling-Test was used as goodness-of-fit test and data distribution shown as histogram.

For the calculation of the reference interval, parametric methods were applied after logarithmic transformation due to non-normal distribution of data. CRP concentration in the healthy dogs ranged between 0.1–22.8 mg/l. The upper one-sided 95% reference limit was 10.8 mg/l (90% CI 7.9–14.9%).

### Classification of diseases

#### Etiology

The diagnosis was made based on the diagnosis of the responsible veterinarians as a conclusion of anamnesis, clinical findings and further diagnostics including laboratory tests and diagnostic imaging. The diseases were classified into the following disease categories: inflammatory, neoplastic (benign/malignant), tissue damage (such as traumatic event or surgery) and “diverse” (mixed diseases or diseases which cannot be categorized). The category “inflammatory disease” was further subdivided into infectious (bacterial, infectious non-bacterial, multiple pathogens), non-infectious and non-classifiable. The diagnosis “bacterial” was based on a positive microbiological result which was considered clinically relevant or a direct positive finding of bacteria in cytological or histological specimens or the clinical response to antibiotics after suspicion of a bacterial infection without diagnostic results indicating another etiology. The category “multiple pathogens” contained variable combinations of viral, bacterial or parasitic disease.

Malignant neoplastic disease was diagnosed by cytology or histopathology or the cytological/histological proof of a metastasis. Neoplastic diseases were further subclassified as epithelial, mesenchymal or round cell neoplasia according to cytological or histological criteria.

If a detailed categorization of the clinical cases was not possible, the categorization was terminated at the last subcategory that could be defined.

#### Affected organ system

Furthermore, the diseases were categorized according to the affected organ system into the following subgroups: skin/subcutis/ear, respiratory tract, heart, gastrointestinal tract, urinary and reproductive tract, musculoskeletal system, central/peripheral nervous system. If several organ systems were affected, the dogs were included in the category “multiple”. The only exception were trauma patients. They were classified as “trauma” even if multiple organ systems were affected.

#### Prognosis

The prognosis was reflected by the 3-months survival rate and the hospitalization rate. The survival rate was investigated in a subpopulation of dogs with documented outcome 3 months after assessment of CRP > 100 mg/l. The decision for hospitalization was made by the owner after consultation of the referring veterinarian, whereby the decisive arguments were not investigated here.

### Statistical analysis

The statistical software programs GraphPad Prism 7.02 Software (GraphPad Software, Inc., La Jolla, USA) and Microsoft Excel® (Microsoft Office 2013, Microsoft Corporation, Redmond, USA) were used for statistical assessment of the obtained data. The level of significance was *p* < 0.05. Normality was assessed using the Shapiro-Wilk Test. As data was not normally distributed, nonparametric methods were applied.

#### Prevalence of CRP > 100 mg/l

Descriptive statistics were applied to describe CRP values > 100 mg/l among laboratory data and patients.

#### Investigation of etiology and affected organ system

Differences between CRP results of the etiological groups and subgroups as well as of the organ systems were assessed with a Kruskal-Wallis-Test. If the subgroups included < 4 dogs, only descriptive statistics were applied.

#### Investigation of prognosis

A Mann Whitney test was used to assess the impact of 3-months survival or hospitalization on the CRP concentration. Comparison of the survival rate in the different disease groups was done with a Chi-square-test. If the subgroups included < 4 dogs, only descriptive statistics were applied.

## Data Availability

The datasets analyzed during the current study are available from the corresponding author on reasonable request.

## Abbreviations

APP: Acute phase protein(s)
CRP: C-reactive protein
